# Supplementary material for: Electrifying passenger road transport in India requires near-term electricity grid decarbonisation
Source: Nat Commun. 2022 Apr 19;13:2095. doi: 10.1038/s41467-022-29620-x (PMC9018792; doi:10.1038/s41467-022-29620-x)
Supplement: Supplementary file 1 — Supplementary Information [file 41467_2022_29620_MOESM1_ESM.pdf]

Supporting Information: raw data and extended plots

Electrifying passenger road transport in India requires near-term electricity grid decarbonisation

Amir F.N. Abdul-Manan[1,2*], Victor Gordillo Zavaleta[3], Avinash Kumar Agarwal[4], Gautam Kalghatgi[5], Amer A. Amer[2]

[1] Strategic Transport Analysis Team, Beijing Research Center, Aramco Asia, Beijing, China

[2] Transport Technologies R&D Division, Saudi Aramco Research & Development Center (R&DC), Dhahran, Saudi Arabia

[3] Aramco Fuel Research Center, Aramco Overseas Company B.V. Paris, France

[4] Engine Research Laboratory, Department of Mechanical Engineering, Indian Institute of Technology Kanpur, Kanpur, India

[5] Consultant Professor, Shanghai Jiao Tong University, Shanghai, China.

[*] Corresponding Author: amir.abdulmanan@aramco.com

Supplementary Figures

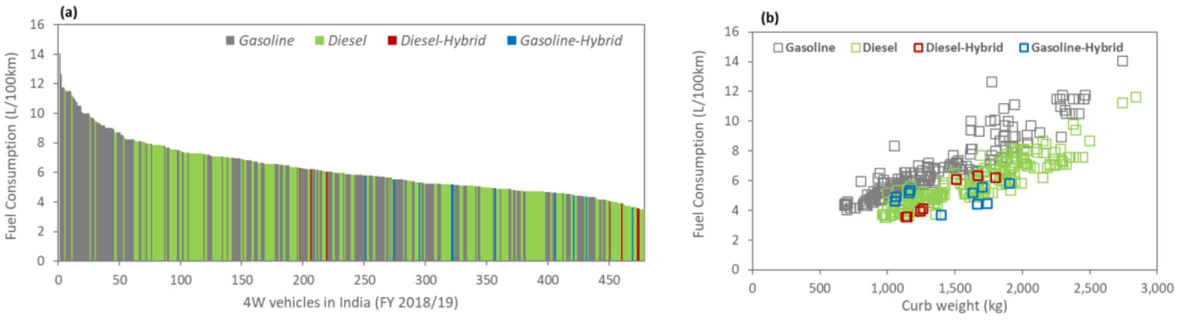

Supplementary Figure 1 Vehicle fuel consumption. (a) Fuel consumption of 478 passenger-road, 4-wheelers in India commercially available during the financial year 2018/19, broken down by fuel and powertrain types. (b) Correlation between fuel consumption and vehicle curb weight by fuel and powertrain types. Data from [1]

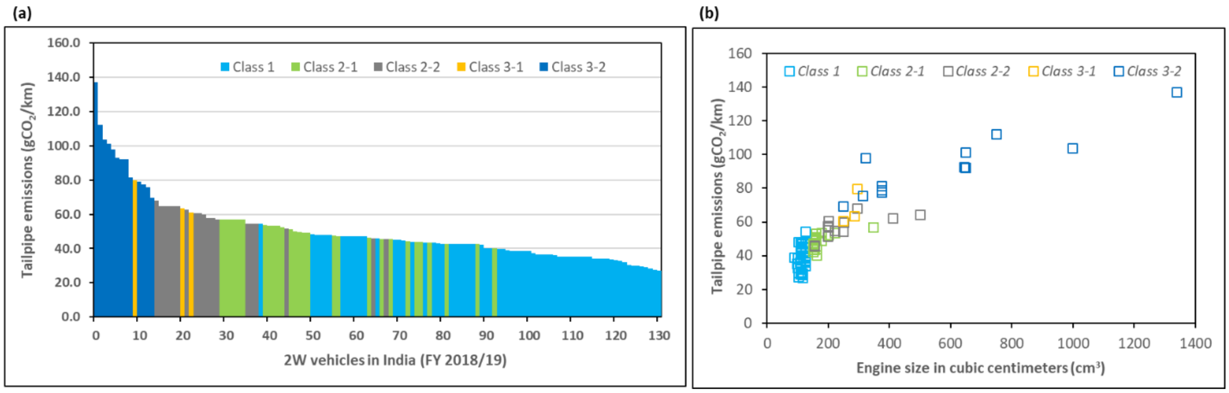

Supplementary Figure 2 Tailpipe emissions of 2-wheelers. (a) Vehicle tailpipe emissions of 131 traditional 2-wheelers, broken down by vehicle class, were commercially available in India in the financial year 2018/19. (b) Correlation between 2-wheeler exhaust CO_2 emissions and engine size by class type. Data from [1]

Saudi Aramco: Public

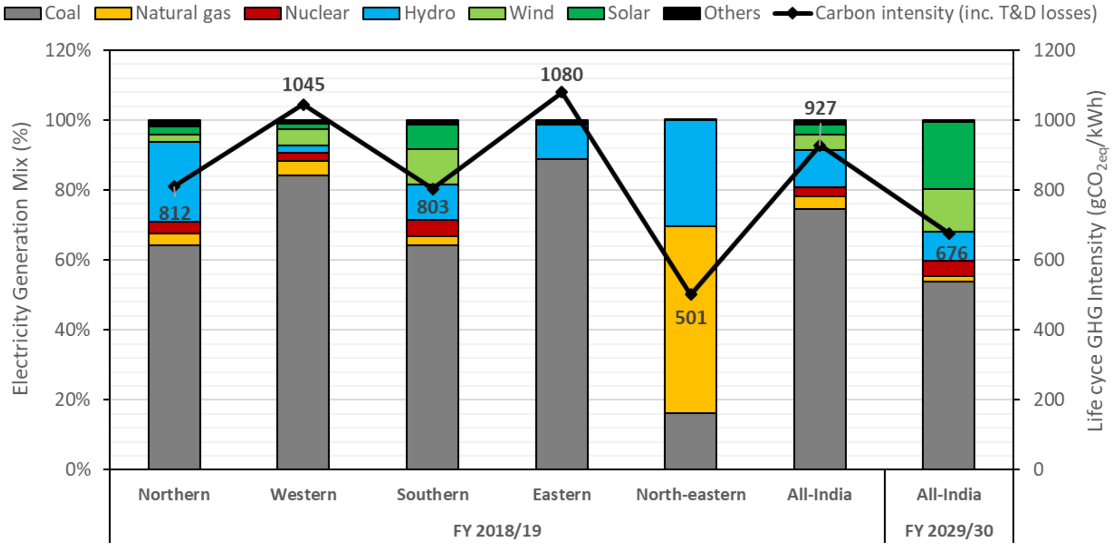

Supplementary Figure 3 Electricity generation in India. Power generation mix in different regions and India-average for the financial year 2018/19 [2], and forecast for 2029/30 based on the CEA [3]. The resulting lifecycle GHG emission intensities include the T&D losses [4].

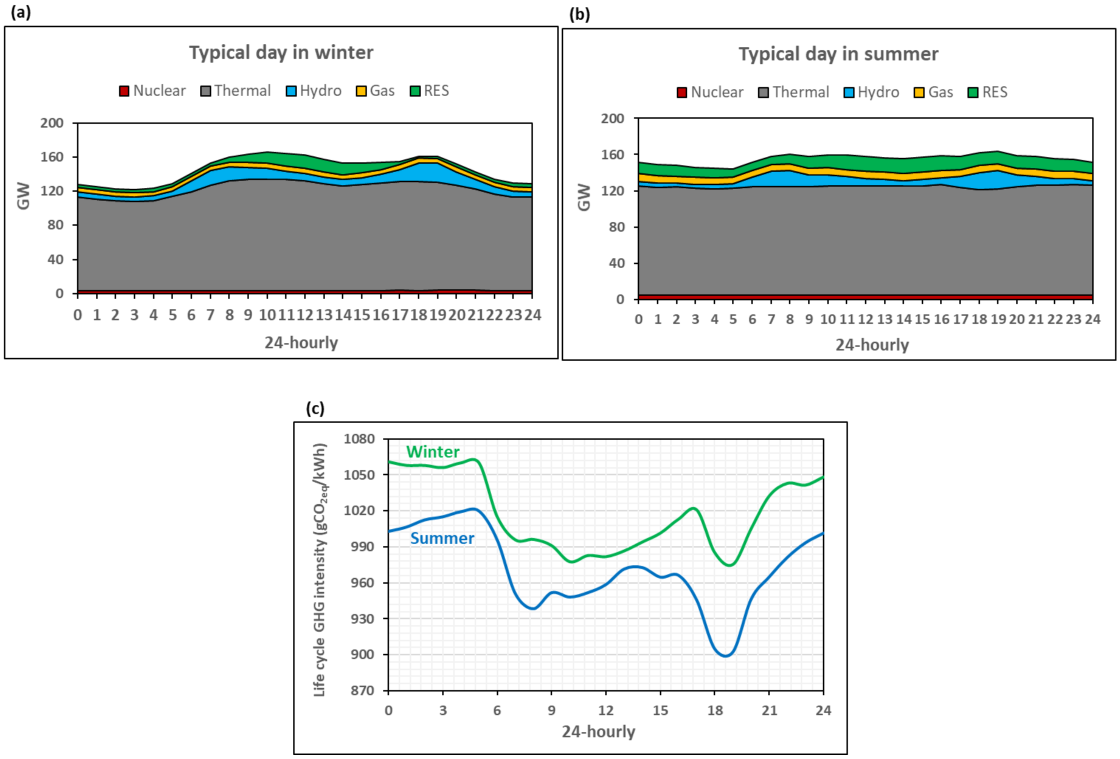

Supplementary Figure 4 Seasonal and hourly power generation profiles. 24-hourly electricity generation mixes on a (a) typical winter day and (b) typical summer day in India in 2018/19 [2]. (c) The 24-hourly life cycle GHG emission intensity profiles for electricity in India in summer and winter, inclusive of T&D losses [4].

Saudi Aramco: Public

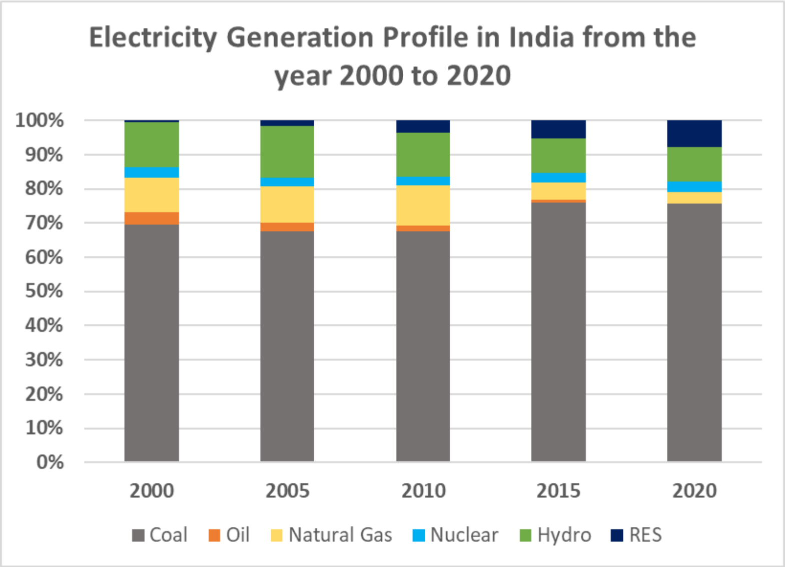

Supplementary Figure 5 Electricity generation profile in India in the last 20 years from 2000 to 2020. Data were derived from the IEA [5] and India's CEA [2].

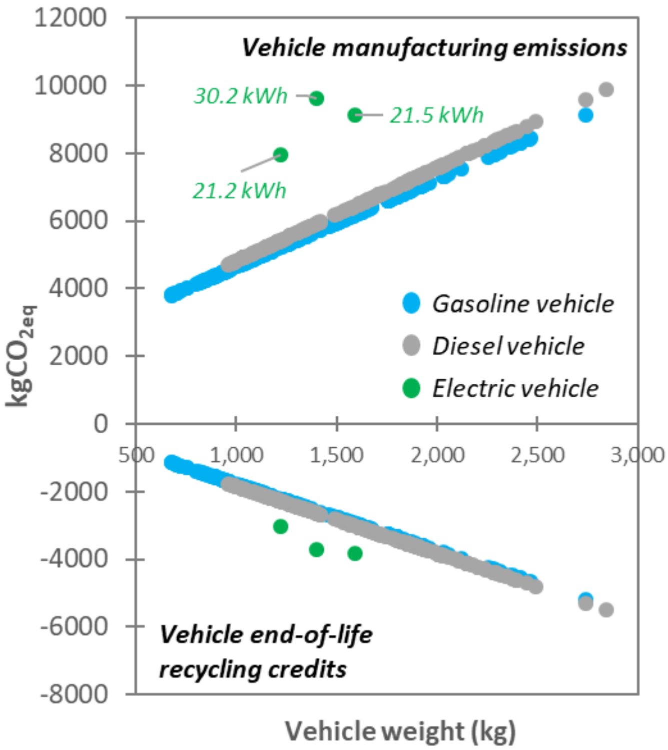

Supplementary Figure 6 Vehicle life cycle emissions. Manufacturing emissions and end-of-life recycling credits for gasoline, diesel and electric vehicles, with different curb weights. Life cycle emissions of electric vehicles also rely heavily on the size of the battery installed (labelled in kWh). Data from Sphera's LCA software and databases (GaBi version 9.2 with 2020 LCI Databases) [6]

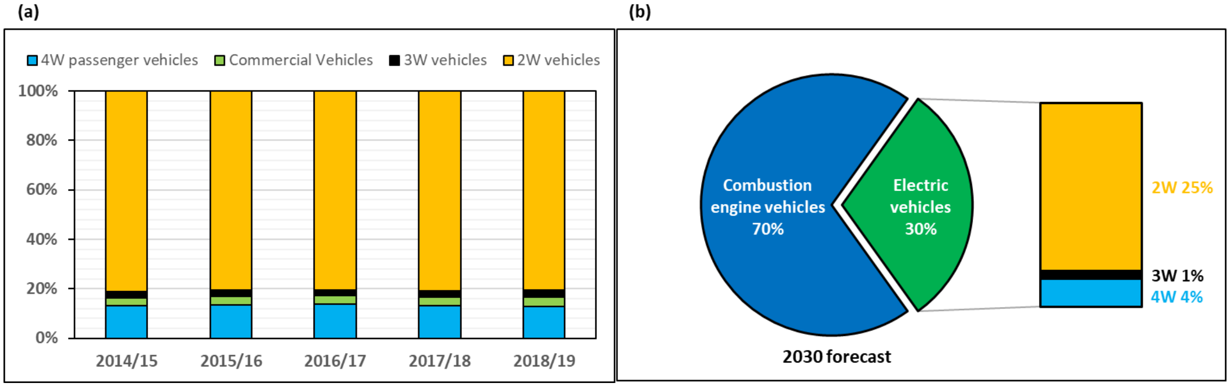

Supplementary Figure 7 Vehicle sales in India. (a) Breakdown of vehicle sales in India from the financial year 2014/15 to 2018/19 [7]. (b) Light-duty vehicle sales forecast for the EV30@30 scenario by the Council on Energy, Environment and Water (CEEW) India [8]. India is projected to exceed 51.2 million new vehicle sales annually by 2030.

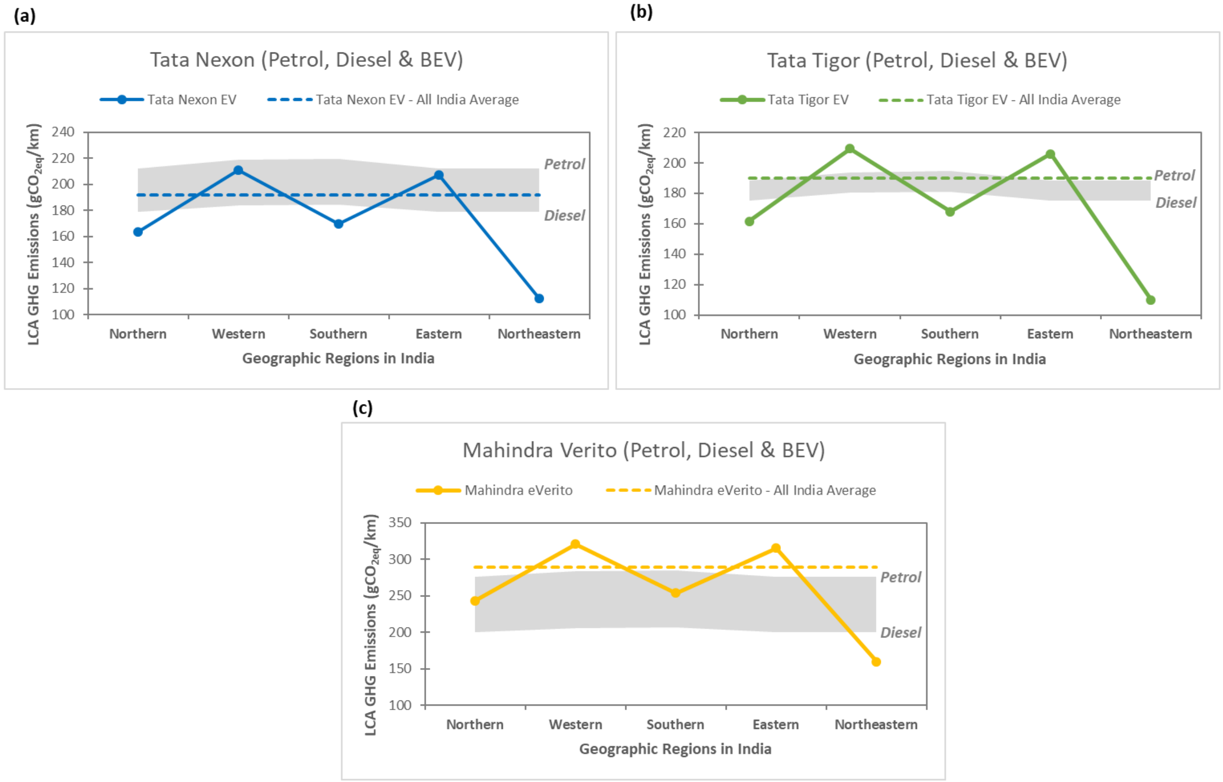

Supplementary Figure 8 Life cycle GHG emissions of (a) Tata Nexon, (b) Tata Tigor, and (c) Mahindra Verito, for three matching powertrains (gasoline spark-ignition, diesel compression-ignition, and battery electric vehicle).

Saudi Aramco: Public

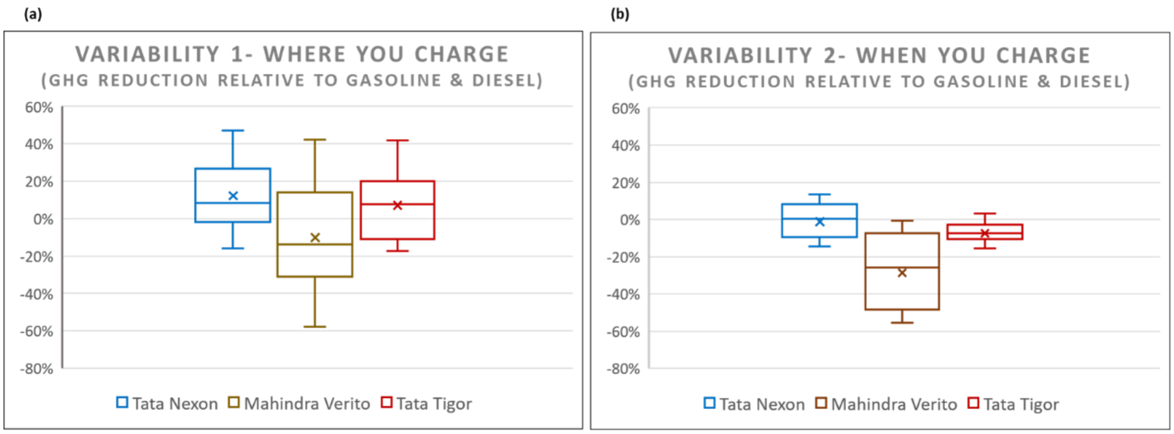

Supplementary Figure 9 Variability in GHG emissions reductions. The life cycle GHG emission benefits of BEVs relative to gasoline and diesel vehicles can vary significantly based on (a) where it is charged and (b) when the charging takes place. (Negative percentages denote that BEVs have higher emissions than conventional vehicles.)

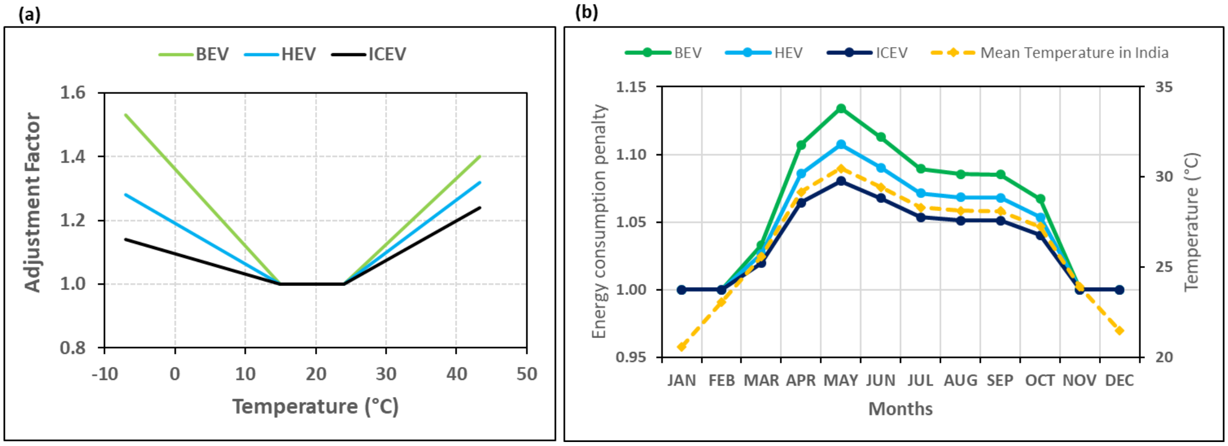

Supplementary Figure 10 Effects of mean ambient temperature on vehicle energy consumption. (a) Correlation between mean ambient temperature and adjustment factors for ICE, HEV, and BEV, plotted by the authors based on Wu et al. [9]. (b) Estimated energy consumption penalty in India based on the official mean ambient temperatures for each month [10].

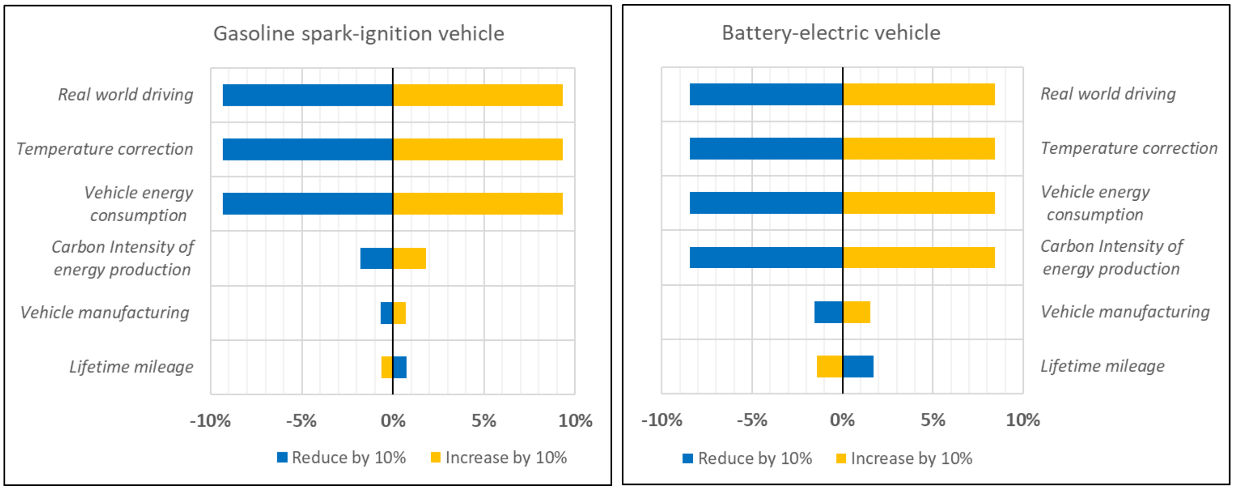

Supplementary Figure 11 Four-wheeler uncertainty analysis. The effects of varying key input parameters by +/- 10% on the overall life cycle GHG emissions.

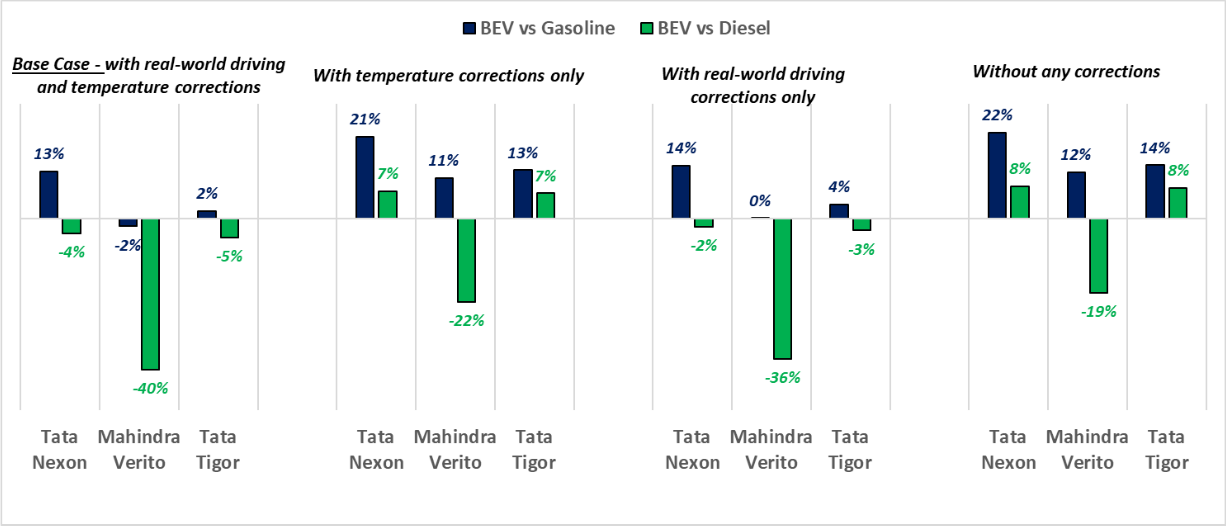

Supplementary Figure 12 GHG reduction potentials of BEV, with and without real-world driving corrections and ambient temperature corrections. Results for all-India average.

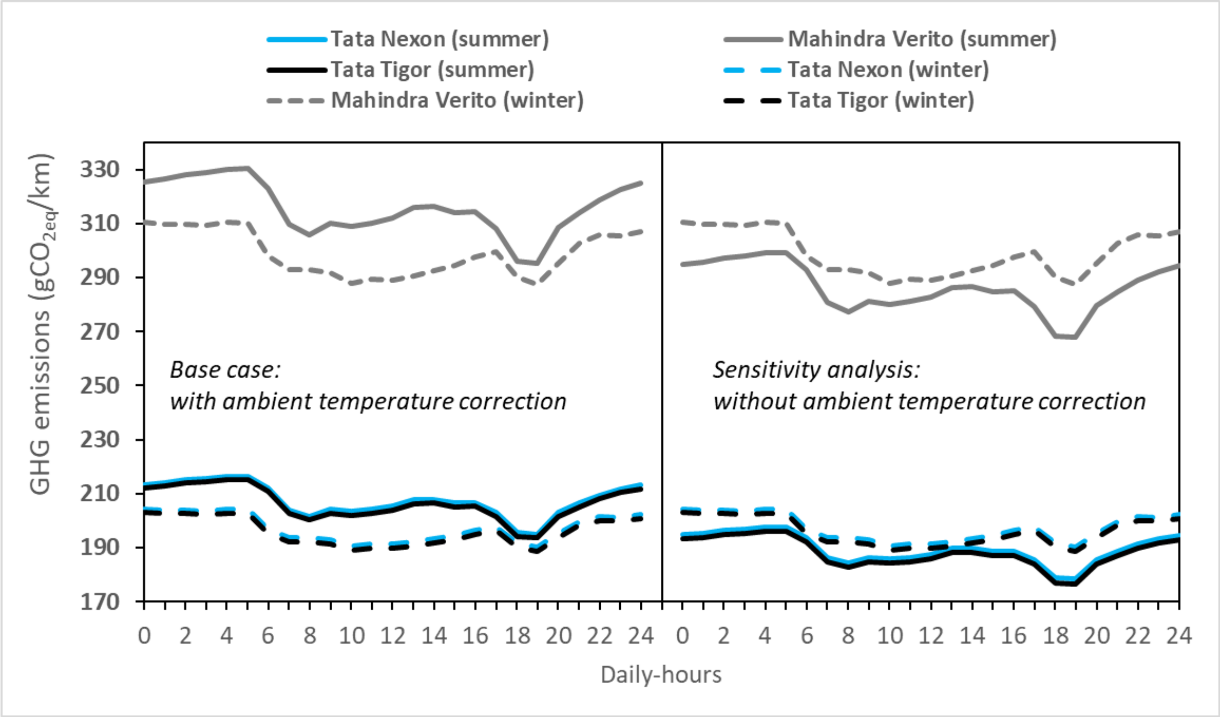

Supplementary Figure 13 Seasonal variation. Effects of ambient temperature penalty on life cycle GHG emissions of BEVs in summer and winter.

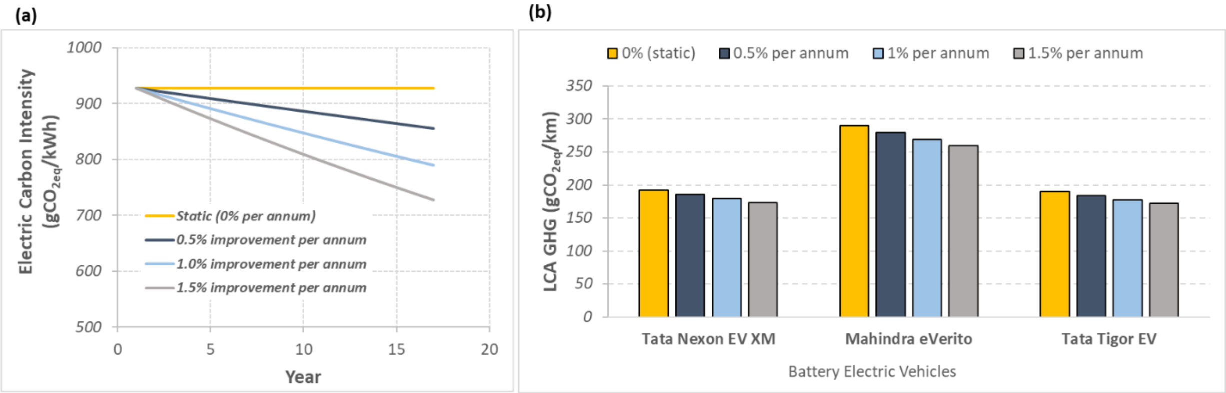

Supplementary Figure 14 The effects of adopting a dynamic carbon intensity profile on the life cycle GHG emissions of BEVs in India. (a) Three emissions improvement rates (0.5%, 1%, and 1.5% per year) were assessed relative to a static electricity base case. (b) The net GHG emissions of BEVs over the vehicle life cycle under the different grid decarbonization scenarios.

(a) Year 2018/19

BEV Electric consumption (Wh/km); rows: Gasoline fuel consumption (L/100km)

Gasoline fuel consumption (L/100km)	90	100	110	120	130	140	150
3.5	123%	134%	146%	157%	169%	180%	191%
4.0	109%	119%	129%	139%	149%	160%	170%
4.5	98%	107%	116%	125%	134%	143%	152%
5.0	89%	97%	105%	114%	122%	130%	138%
5.5	81%	89%	97%	104%	112%	119%	127%
6.0	75%	82%	89%	96%	103%	110%	117%
6.5	70%	76%	83%	89%	95%	102%	108%
7.0	65%	71%	77%	83%	89%	95%	101%

(b) Year 2029/30

Base Case: Electric CI = 676 gCO_{2eq}/kWh

BEV Electric consumption (Wh/km); rows: Gasoline fuel consumption (L/100km)

Gasoline fuel consumption (L/100km)	90	100	110	120	130	140	150
3.5	95%	104%	112%	120%	129%	137%	145%
4.0	85%	92%	99%	107%	114%	121%	129%
4.5	76%	83%	89%	96%	102%	109%	116%
5.0	69%	75%	81%	87%	93%	99%	105%
5.5	63%	69%	74%	80%	85%	91%	96%
6.0	58%	63%	68%	73%	78%	84%	89%
6.5	54%	59%	63%	68%	73%	77%	82%
7.0	50%	55%	59%	63%	68%	72%	77%

Base Case minus 15%: Electric CI = 575 gCO_{2eq}/kWh

BEV Electric consumption (Wh/km); rows: Gasoline fuel consumption (L/100km)

Gasoline fuel consumption (L/100km)	90	100	110	120	130	140	150
3.5	84%	91%	98%	105%	112%	119%	126%
4.0	75%	81%	87%	93%	100%	106%	112%
4.5	67%	73%	78%	84%	90%	95%	101%
5.0	61%	66%	71%	76%	81%	86%	91%
5.5	56%	60%	65%	70%	74%	79%	84%
6.0	51%	56%	60%	64%	69%	73%	77%
6.5	48%	52%	56%	60%	64%	68%	72%
7.0	44%	48%	52%	56%	59%	63%	67%

Base Case plus 15%: Electric CI = 778 gCO_{2eq}/kWh

BEV Electric consumption (Wh/km); rows: Gasoline fuel consumption (L/100km)

Gasoline fuel consumption (L/100km)	90	100	110	120	130	140	150
3.5	107%	116%	126%	135%	145%	154%	164%
4.0	95%	103%	111%	120%	128%	137%	145%
4.5	85%	93%	100%	108%	115%	123%	130%
5.0	77%	84%	91%	98%	105%	112%	118%
5.5	71%	77%	83%	89%	96%	102%	108%
6.0	65%	71%	77%	83%	88%	94%	100%
6.5	60%	66%	71%	77%	82%	87%	93%
7.0	56%	61%	66%	71%	76%	81%	86%

Supplementary Figure 15 GHG emissions difference between BEV and gasoline spark-ignition with varying fuel and electricity consumption, based on all-India power generation profiles in the financial year (a) 2018/19, and (b) 2029/30, for a 4W vehicle with a curb weight of 1237kg. 100% = total BEV emissions equal total emissions from gasoline vehicles, with the colour red denoting that the gasoline vehicle is better, and green means that the BEV has a lower emission. For the financial year 2029/2030, three power generation scenarios were assessed: a base case with a projected electricity carbon intensity of 676 gCO_{2eq}/kWh and two sensitivity cases with +/- 15%.

Saudi Aramco: Public

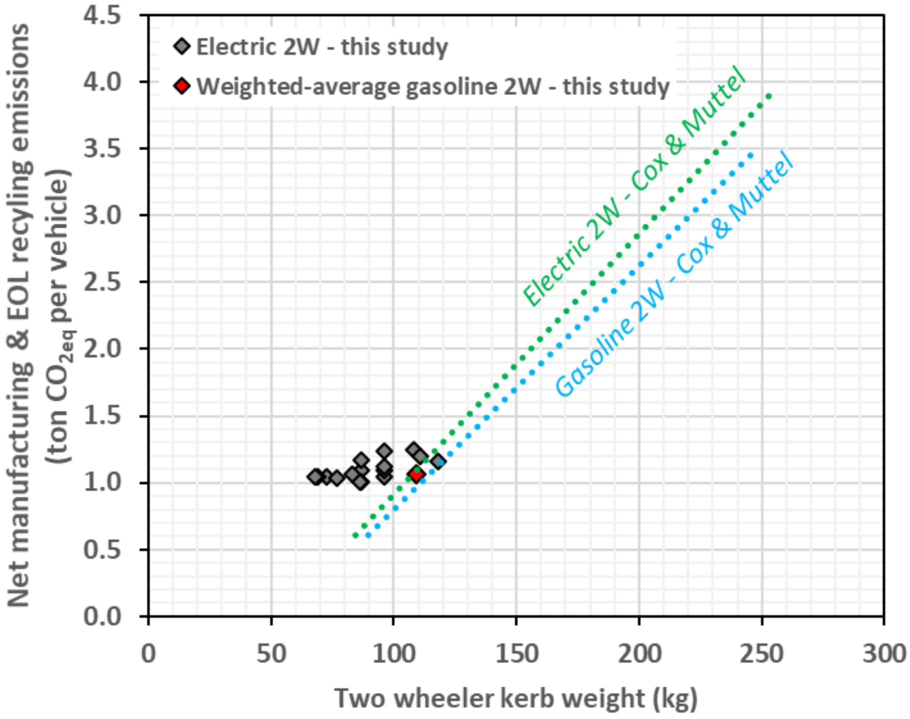

Supplementary Figure 16 Manufacturing emissions for 2W. Net manufacturing and end-of-life recycling of gasoline and electric two-wheelers. A comparison between the estimates used in this study and the literature values for European 2W was estimated by Cox & Muttel [11]. The electric 2W in this study had a battery size in the range of 1.0 to 3.3 kWh.

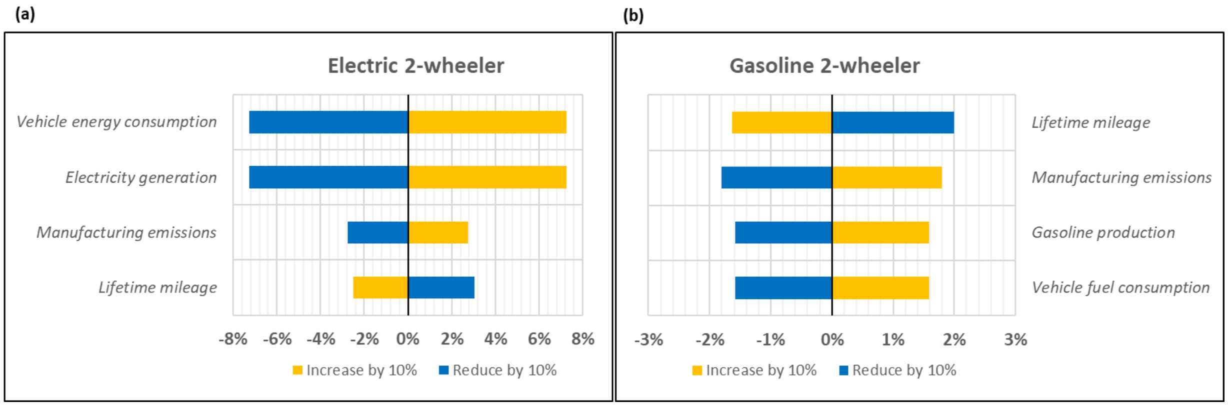

Supplementary Figure 17 Two-wheeler uncertainty analysis. The effects of varying key input parameters by +/- 10% on the overall life cycle GHG emissions of (a) electric 2W and (b) gasoline 2W.

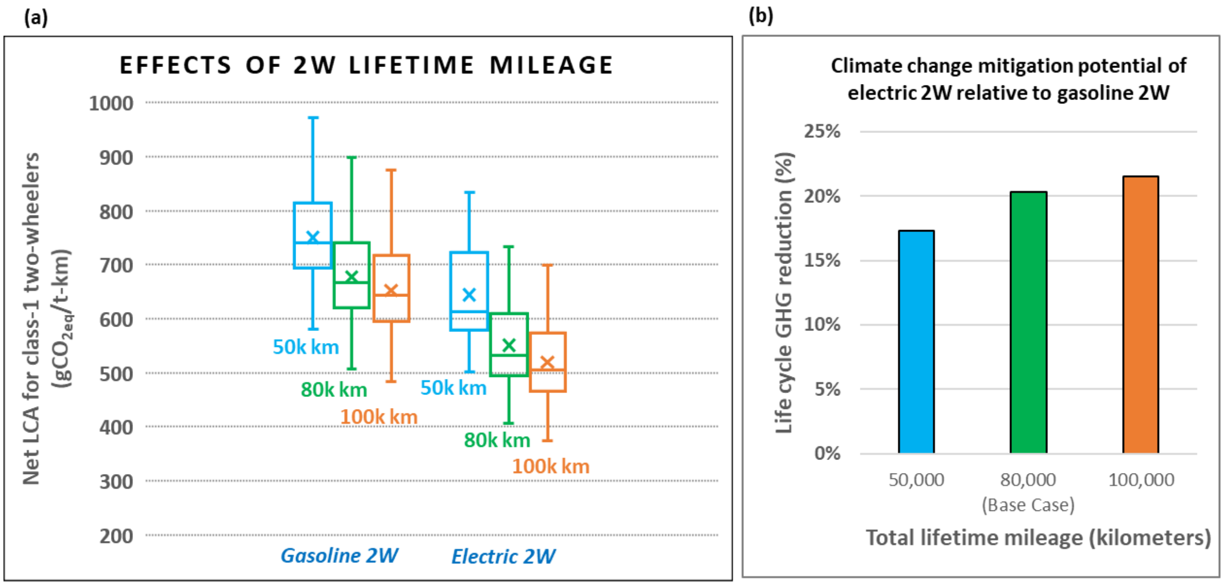

Supplementary Figure 18 Effects of total lifetime mileage on (a) the life cycle GHG emissions of gasoline and electric 2-wheelers, and (b) the median GHG reduction potential of electric 2-wheelers.

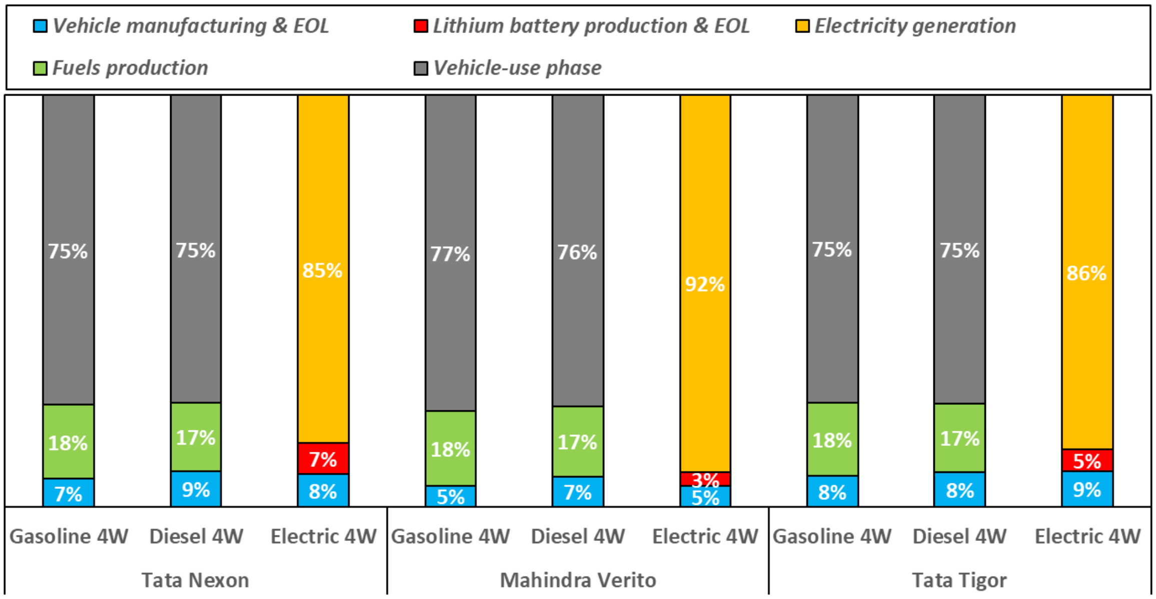

Supplementary Figure 19 Lifecycle GHG emissions breakdown for gasoline, diesel and electric 4W using the average electricity mix in India in 2018/19.

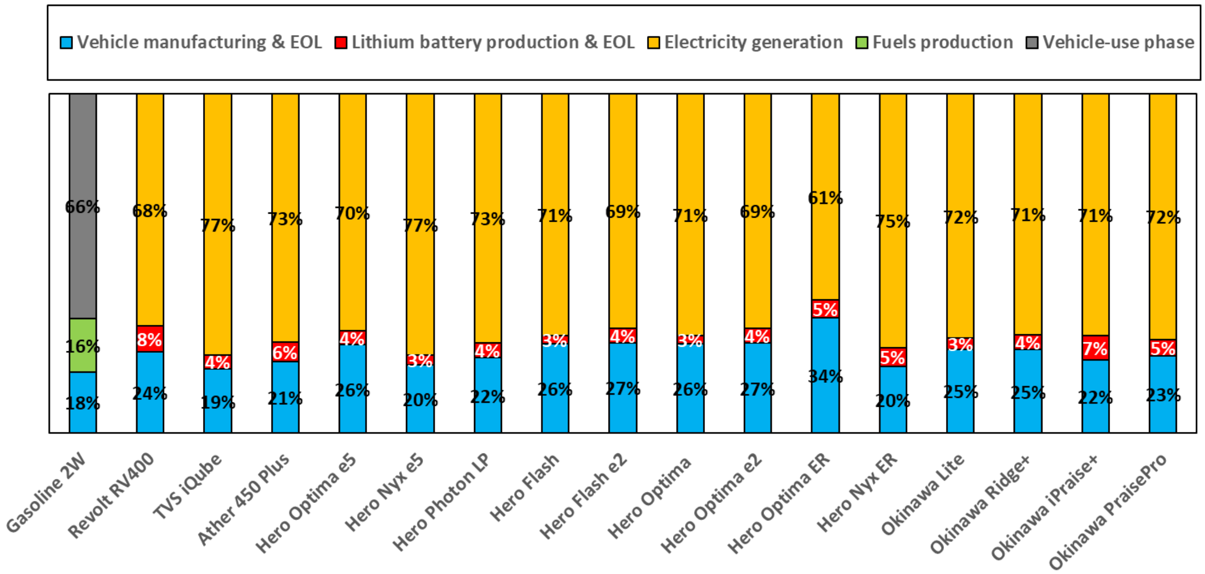

Supplementary Figure 20 Life cycle GHG emissions breakdown for gasoline and electric 2W using the average electricity mix in India in 2018/19.

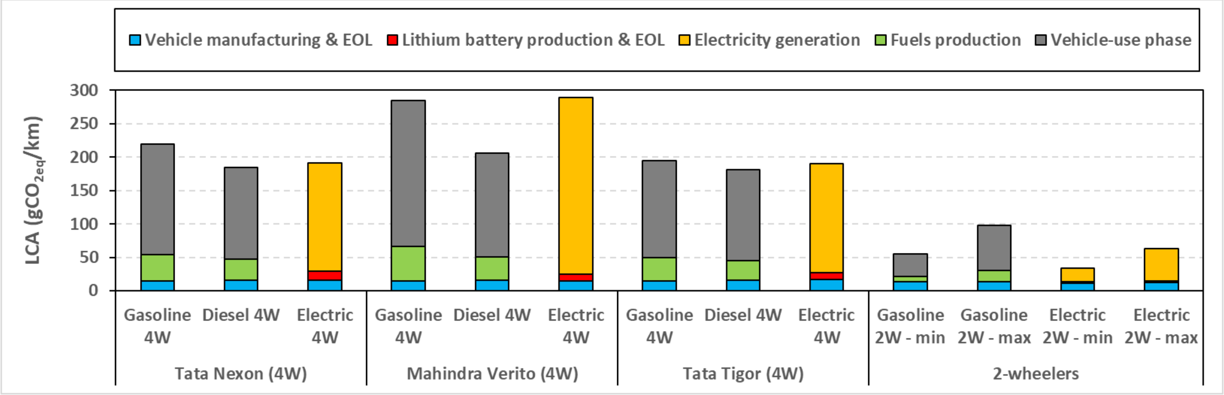

Supplementary Figure 21 A comparison of the life cycle GHG emissions contributions from 4W and 2W, in gCO_{2eq}/km, using the average electricity mix in India in 2018/19.

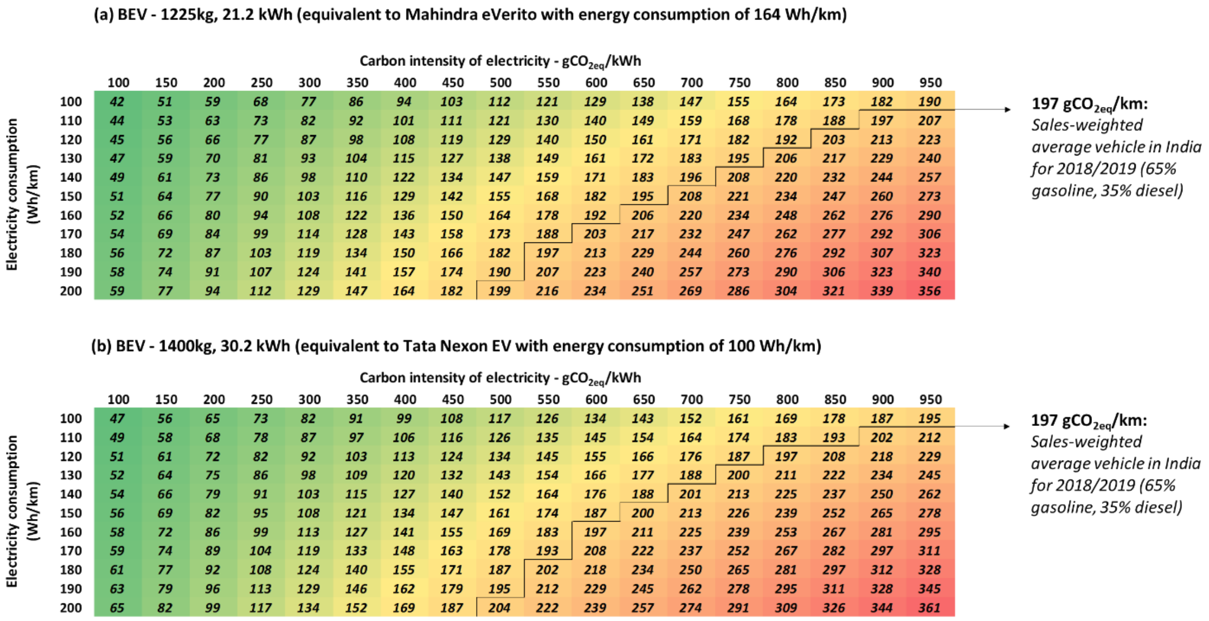

Supplementary Figure 22 Life cycle GHG emissions of 4W BEVs (in gCO_{2eq}/km) with different electricity consumptions and powered by varying electricity carbon intensity for (a) a BEV with 1225kg curb weight and equipped with a 21.2 kWh battery, equivalent to a Mahindra eVerito, and (b) a BEV with 1400kg curb weight and equipped with a 30.2 kWh battery, equivalent to a Tata Nexon EV. The sales-weighted average vehicle in India in 2018/19, comprising 65% gasoline and 35% diesel cars, has a curb weight of 1078 kg and tailpipe emission of 121.9 gCO_2/km [12], resulting in an estimated total life cycle GHG emission of 197gCO_{2eq}/km. The current grid mix has a carbon intensity of 927 gCO_{2eq}/kWh.

Supplementary Tables

Supplementary Table 1 Raw data on tailpipe emissions and fuel economy of 478 passenger road 4W vehicles in India in 2018/19 [1].

Count	Curb Weight (kg)	Engine size (cm^3)	Emission Category	Fuel Type	Fuel Economy (km/L)	Tailpipe CO_2 (g/km)	Fuel Consumption (L/100km)
1	1,905	2,494	BS IV	Hybrid Gasoline	17.2	138.0	5.8
2	1,705	2,494	BS IV	Hybrid Gasoline	18.0	132.0	5.6
3	1,170	1,462	BS IV	Hybrid Gasoline	18.7	126.9	5.3
4	1,635	2,494	BS IV	Hybrid Gasoline	19.3	123.0	5.2
5	1,160	1,462	BS IV	Hybrid Gasoline	19.3	122.6	5.2
6	1,065	1,462	BS IV	Hybrid Gasoline	20.3	116.9	4.9
7	1,055	1,462	BS IV	Hybrid Gasoline	21.6	110.0	4.6

8	1,740	2,487	BS IV	Hybrid Gasoline	22.4	106.0	4.5
9	1,665	2,487	BS IV	Hybrid Gasoline	22.8	104.0	4.4
10	1,400	1,798	BS IV	Hybrid Gasoline	27.3	87.0	3.7
11	1,670	2,523	BS IV	Hybrid Diesel	15.8	167.9	6.3
12	1,800	2,523	BS IV	Hybrid Diesel	16.1	164.8	6.2
13	1,510	1,493	BS IV	Hybrid Diesel	16.5	160.5	6.1
14	1,265	1,248	BS IV	Hybrid Diesel	24.5	108.0	4.1
15	1,245	1,248	BS IV	Hybrid Diesel	25.5	104.0	3.9
16	1,145	1,248	BS IV	Hybrid Diesel	28.1	94.3	3.6
17	1,135	1,248	BS IV	Hybrid Diesel	28.1	94.3	3.6
18	2,740	5,663	BS IV	Gasoline	7.1	332.0	14.1
19	1,770	4,951	BS IV	Gasoline	7.9	300.8	12.7
20	2,465	2,996	BS IV	Gasoline	8.5	280.0	11.8
21	2,295	3,982	BS VI	Gasoline	8.5	280.0	11.8
22	2,457	2,995	BS IV	Gasoline	8.7	273.0	11.5
23	2,415	2,995	BS IV	Gasoline	8.7	273.0	11.5
24	2,375	2,995	BS IV	Gasoline	8.7	273.0	11.5
25	2,278	2,995	BS IV	Gasoline	8.7	272.8	11.5
26	2,255	2,995	BS IV	Gasoline	8.7	272.8	11.5
27	1,939	2,996	BS IV	Gasoline	9.0	262.4	11.1
28	2,280	2,996	BS IV	Gasoline	9.1	262.0	11.0
29	1,860	2,179	BS IV	Gasoline	9.2	258.0	10.9
30	2,312	1,997	BS IV	Gasoline	9.3	255.9	10.8
31	2,415	2,995	BS IV	Gasoline	9.5	248.9	10.5
32	2,366	2,995	BS IV	Gasoline	9.5	248.9	10.5
33	2,322	2,995	BS IV	Gasoline	9.5	248.9	10.5
34	1,793	2,996	BS VI	Gasoline	9.9	240.0	10.1
35	1,760	2,694	BS IV	Gasoline	10.0	238.0	10.0
36	1,760	2,694	BS IV	Gasoline	10.0	238.0	10.0
37	1,760	2,694	BS IV	Gasoline	10.0	238.0	10.0
38	1,760	2,694	BS IV	Gasoline	10.0	238.0	10.0
39	1,615	2,996	BS IV	Gasoline	10.0	237.0	10.0
40	1,895	2,694	BS IV	Gasoline	10.0	236.0	10.0
41	2,060	2,996	BS IV	Gasoline	10.3	230.0	9.7
42	1,871	1,991	BS IV	Gasoline	10.3	230.0	9.7
43	1,925	2,694	BS IV	Gasoline	10.4	229.0	9.6
44	1,616	2,979	BS VI	Gasoline	10.6	223.0	9.4
45	1,678	2,979	BS IV	Gasoline	10.7	222.0	9.3
46	1,678	2,979	BS IV	Gasoline	10.7	222.0	9.3
47	2,125	2,979	BS IV	Gasoline	10.8	220.0	9.3
48	1,800	2,694	BS IV	Gasoline	10.9	217.0	9.2
49	1,800	2,694	BS IV	Gasoline	10.9	217.0	9.2
50	1,800	2,694	BS IV	Gasoline	10.9	217.0	9.2

Saudi Aramco: Public

51	2,031	1,997	BS IV	Gasoline	11.0	216.0	9.1
52	1,943	1,997	BS IV	Gasoline	11.1	213.8	9.0
53	1,929	1,997	BS IV	Gasoline	11.1	213.8	9.0
54	1,929	1,997	BS IV	Gasoline	11.1	213.8	9.0
55	1,927	1,997	BS IV	Gasoline	11.1	213.8	9.0
56	1,927	1,997	BS IV	Gasoline	11.1	213.8	9.0
57	1,860	1,998	BS VI	Gasoline	11.1	213.0	9.0
58	2,286	2,998	BS VI	Gasoline	11.2	211.0	8.9
59	1,860	1,997	BS IV	Gasoline	11.5	204.6	8.7
60	1,833	1,997	BS IV	Gasoline	11.5	204.6	8.7
61	1,831	1,997	BS IV	Gasoline	11.5	204.6	8.7
62	2,037	1,997	BS IV	Gasoline	11.7	204.2	8.5
63	2,032	1,997	BS IV	Gasoline	11.7	204.2	8.5
64	1,050	1,298	BS IV	Gasoline	12.0	197.6	8.3
65	1,930	1,997	BS IV	Gasoline	12.1	196.8	8.3
66	1,930	1,997	BS IV	Gasoline	12.1	196.8	8.3
67	1,918	1,997	BS IV	Gasoline	12.1	196.8	8.3
68	1,918	1,997	BS IV	Gasoline	12.1	196.8	8.3
69	1,916	1,997	BS IV	Gasoline	12.1	196.8	8.3
70	1,916	1,997	BS IV	Gasoline	12.1	196.8	8.3
71	1,874	1,997	BS IV	Gasoline	12.1	196.8	8.3
72	1,628	1,997	BS IV	Gasoline	12.3	193.1	8.1
73	1,615	1,997	BS IV	Gasoline	12.3	193.1	8.1
74	1,965	2,998	BS VI	Gasoline	12.4	191.0	8.1
75	1,800	1,991	BS VI	Gasoline	12.5	190.0	8.0
76	1,926	1,997	BS IV	Gasoline	12.6	188.2	7.9
77	1,610	2,998	BS VI	Gasoline	12.8	185.0	7.8
78	1,514	1,999	BS IV	Gasoline	13.9	183.6	7.2
79	1,485	1,999	BS IV	Gasoline	13.0	182.4	7.7
80	1,770	1,991	BS VI	Gasoline	13.0	182.0	7.7
81	1,756	1,997	BS IV	Gasoline	13.5	178.1	7.4
82	1,881	1,998	BS VI	Gasoline	13.3	178.0	7.5
83	1,843	1,998	BS VI	Gasoline	13.3	178.0	7.5
84	1,786	1,997	BS IV	Gasoline	13.5	175.9	7.4
85	1,525	1,991	BS IV	Gasoline	13.6	174.0	7.4
86	1,509	1,998	BS IV	Gasoline	13.8	172.0	7.2
87	1,303	1,498	BS IV	Gasoline	13.9	171.0	7.2
88	1,762	1,998	BS VI	Gasoline	14.0	170.0	7.1
89	1,510	1,991	BS IV	Gasoline	14.0	170.0	7.1
90	1,465	1,396	BS IV	Gasoline	14.1	168.8	7.1
91	1,227	1,498	BS IV	Gasoline	14.2	167.0	7.0
92	1,295	1,498	BS IV	Gasoline	15.9	166.7	6.3
93	1,583	1,998	BS IV	Gasoline	14.3	165.3	7.0

94	1,457	1,396	BS IV	Gasoline	14.3	165.3	7.0
95	1,480	1,998	BS VI	Gasoline	14.4	165.0	6.9
96	1,295	1,798	BS IV	Gasoline	14.4	165.0	6.9
97	1,295	1,798	BS IV	Gasoline	14.4	165.0	6.9
98	1,295	1,798	BS IV	Gasoline	14.4	165.0	6.9
99	1,545	1,997	BS VI	Gasoline	14.4	164.9	6.9
100	1,418	1,984	BS IV	Gasoline	14.5	164.0	6.9
101	1,300	1,999	BS IV	Gasoline	14.6	163.4	6.8
102	1,338	1,999	BS IV	Gasoline	14.6	162.9	6.8
103	1,298	1,497	BS IV	Gasoline	14.6	162.2	6.8
104	1,494	1,798	BS IV	Gasoline	14.6	162.0	6.8
105	1,348	1,591	BS IV	Gasoline	14.8	160.2	6.8
106	1,600	1,497	BS IV	Gasoline	14.8	160.0	6.8
107	1,540	1,798	BS IV	Gasoline	14.8	160.0	6.8
108	1,258	1,498	BS IV	Gasoline	15.0	158.1	6.7
109	1,670	1,998	BS VI	Gasoline	15.0	158.0	6.7
110	1,376	1,798	BS IV	Gasoline	15.1	157.0	6.6
111	1,660	1,997	BS IV	Gasoline	15.2	155.1	6.6
112	1,170	1,598	BS IV	Gasoline	15.3	155.0	6.5
113	1,135	1,193	BS IV	Gasoline	15.3	155.0	6.5
114	1,125	1,193	BS IV	Gasoline	15.3	155.0	6.5
115	940	1,196	BS IV	Gasoline	15.4	154.2	6.5
116	940	1,196	BS IV	Gasoline	15.4	154.2	6.5
117	1,235	1,497	BS IV	Gasoline	15.4	154.0	6.5
118	1,565	1,998	BS VI	Gasoline	15.7	151.0	6.4
119	1,655	1,997	BS IV	Gasoline	15.2	150.9	6.6
120	1,625	1,997	BS IV	Gasoline	15.2	150.9	6.6
121	1,318	1,591	BS IV	Gasoline	15.8	150.1	6.3
122	1,175	1,591	BS IV	Gasoline	15.9	149.0	6.3
123	1,137	1,598	BS IV	Gasoline	15.9	149.0	6.3
124	1,238	1,497	BS IV	Gasoline	16.0	148.2	6.3
125	1,140	1,598	BS IV	Gasoline	16.0	148.0	6.3
126	1,606	1,998	BS VI	Gasoline	16.1	147.0	6.2
127	1,310	1,798	BS IV	Gasoline	16.1	147.0	6.2
128	1,310	1,798	BS IV	Gasoline	16.1	147.0	6.2
129	928	1,196	BS IV	Gasoline	16.2	146.4	6.2
130	928	1,196	BS IV	Gasoline	16.2	146.4	6.2
131	1,103	1,497	BS IV	Gasoline	16.3	145.7	6.1
132	1,078	1,497	BS IV	Gasoline	16.3	145.2	6.1
133	1,300	1,799	BS VI	Gasoline	16.5	144.2	6.1
134	1,211	1,497	BS IV	Gasoline	16.5	144.0	6.1
135	1,268	1,799	BS VI	Gasoline	16.5	143.8	6.1
136	970	1,198	BS IV	Gasoline	16.5	143.7	6.1

Saudi Aramco: Public

137	1,350	1,998	BS VI	Gasoline	16.7	142.0	6.0
138	1,307	1,198	BS IV	Gasoline	16.7	142.0	6.0
139	1,143	1,591	BS IV	Gasoline	16.7	142.0	6.0
140	1,252	1,198	BS IV	Gasoline	16.7	141.7	6.0
141	800	796	BS IV	Gasoline	16.8	141.2	6.0
142	1,093	1,197	BS IV	Gasoline	17.0	139.7	5.9
143	1,352	1,198	BS IV	Gasoline	17.0	139.5	5.9
144	1,185	1,373	BS IV	Gasoline	17.0	139.2	5.9
145	1,020	1,498	BS IV	Gasoline	17.0	139.2	5.9
146	975	1,496	BS IV	Gasoline	17.1	139.0	5.8
147	975	1,496	BS IV	Gasoline	17.1	139.0	5.8
148	975	1,496	BS IV	Gasoline	17.1	139.0	5.8
149	975	1,496	BS IV	Gasoline	17.1	139.0	5.8
150	965	1,496	BS IV	Gasoline	17.1	139.0	5.8
151	965	1,496	BS IV	Gasoline	17.1	139.0	5.8
152	1,109	1,197	BS IV	Gasoline	17.1	138.4	5.8
153	1,120	1,496	BS IV	Gasoline	17.2	138.0	5.8
154	1,105	1,198	BS IV	Gasoline	17.2	137.7	5.8
155	1,265	1,998	BS VI	Gasoline	17.3	137.0	5.8
156	1,260	1,395	BS IV	Gasoline	17.3	137.0	5.8
157	1,237	1,198	BS IV	Gasoline	17.3	137.0	5.8
158	1,054	1,197	BS IV	Gasoline	17.4	136.5	5.7
159	1,093	1,197	BS IV	Gasoline	17.4	136.4	5.7
160	1,088	1,497	BS IV	Gasoline	17.4	136.3	5.7
161	1,023	1,197	BS IV	Gasoline	17.5	135.7	5.7
162	1,180	1,373	BS IV	Gasoline	17.5	135.5	5.7
163	1,104	1,199	BS IV	Gasoline	17.5	135.5	5.7
164	1,067	1,499	BS IV	Gasoline	17.5	135.5	5.7
165	1,125	1,198	BS IV	Gasoline	17.5	135.4	5.7
166	1,020	1,197	BS IV	Gasoline	17.6	135.0	5.7
167	1,275	999	BS IV	Gasoline	17.6	134.9	5.7
168	1,103	1,591	BS IV	Gasoline	17.7	134.0	5.6
169	1,107	1,497	BS IV	Gasoline	18.0	131.7	5.6
170	1,135	1,496	BS IV	Gasoline	18.1	131.0	5.5
171	955	1,197	BS IV	Gasoline	18.1	131.0	5.5
172	955	1,197	BS IV	Gasoline	18.1	131.0	5.5
173	955	1,197	BS IV	Gasoline	18.1	131.0	5.5
174	955	1,197	BS IV	Gasoline	18.1	131.0	5.5
175	955	1,197	BS IV	Gasoline	18.1	131.0	5.5
176	1,174	1,197	BS IV	Gasoline	18.1	130.9	5.5
177	1,087	1,197	BS IV	Gasoline	18.2	130.5	5.5
178	1,044	1,199	BS IV	Gasoline	18.2	130.3	5.5
179	1,030	1,199	BS IV	Gasoline	18.4	129.0	5.4

180	930	1,198	BS IV	Gasoline	18.5	128.2	5.4
181	935	1,197	BS IV	Gasoline	18.9	126.3	5.3
182	1,030	999	BS IV	Gasoline	18.8	126.2	5.3
183	1,030	1,199	BS IV	Gasoline	19.0	125.0	5.3
184	1,025	1,199	BS IV	Gasoline	19.0	125.0	5.3
185	894	1,198	BS IV	Gasoline	19.0	125.0	5.3
186	1,066	1,199	BS IV	Gasoline	19.0	124.8	5.3
187	1,035	1,197	BS IV	Gasoline	19.0	124.8	5.3
188	945	1,199	BS IV	Gasoline	19.0	124.8	5.3
189	1,046	1,194	BS IV	Gasoline	19.0	124.7	5.3
190	956	1,197	BS IV	Gasoline	19.1	124.2	5.2
191	1,084	1,368	BS IV	Gasoline	19.1	124.0	5.2
192	1,035	1,373	BS IV	Gasoline	19.1	124.0	5.2
193	1,041	1,194	BS IV	Gasoline	19.4	122.5	5.2
194	1,050	999	BS IV	Gasoline	19.4	122.1	5.2
195	969	1,198	BS IV	Gasoline	19.4	122.1	5.2
196	1,003	1,197	BS IV	Gasoline	19.4	122.0	5.2
197	924	1,199	BS IV	Gasoline	19.5	121.6	5.1
198	1,017	1,199	BS IV	Gasoline	19.5	121.5	5.1
199	1,018	1,197	BS IV	Gasoline	19.5	121.0	5.1
200	907	1,198	BS IV	Gasoline	19.7	120.4	5.1
201	882	1,198	BS IV	Gasoline	19.8	119.6	5.1
202	846	1,198	BS IV	Gasoline	19.8	119.6	5.1
203	901	1,086	BS IV	Gasoline	20.3	116.8	4.9
204	898	1,086	BS IV	Gasoline	20.3	116.8	4.9
205	1,021	1,194	BS IV	Gasoline	20.4	116.3	4.9
206	895	998	BS IV	Gasoline	20.5	115.6	4.9
207	890	998	BS IV	Gasoline	20.5	115.6	4.9
208	1,030	1,373	BS IV	Gasoline	20.7	114.4	4.8
209	935	1,197	BS IV	Gasoline	20.8	114.0	4.8
210	940	1,197	BS IV	Gasoline	20.9	113.7	4.8
211	870	1,197	BS IV	Gasoline	20.9	113.5	4.8
212	860	1,197	BS IV	Gasoline	20.9	113.5	4.8
213	950	998	BS IV	Gasoline	21.1	112.4	4.7
214	950	998	BS IV	Gasoline	21.1	112.4	4.7
215	935	1,197	BS IV	Gasoline	21.4	110.8	4.7
216	910	1,197	BS IV	Gasoline	21.4	110.8	4.7
217	890	1,197	BS IV	Gasoline	21.4	110.8	4.7
218	890	1,197	BS IV	Gasoline	21.4	110.8	4.7
219	845	1,197	BS IV	Gasoline	21.5	110.3	4.7
220	835	1,197	BS IV	Gasoline	21.5	110.3	4.7
221	704	999	BS IV	Gasoline	21.7	109.3	4.6
222	895	1,197	BS IV	Gasoline	22.0	107.8	4.5

223	890	1,197	BS IV	Gasoline	22.0	107.8	4.5
224	885	1,197	BS IV	Gasoline	22.0	107.8	4.5
225	880	1,197	BS IV	Gasoline	22.0	107.8	4.5
226	825	998	BS IV	Gasoline	22.5	105.4	4.4
227	820	998	BS IV	Gasoline	22.5	105.4	4.4
228	705	999	BS IV	Gasoline	22.5	105.4	4.4
229	682	999	BS IV	Gasoline	22.5	105.4	4.4
230	694	799	BS IV	Gasoline	22.7	104.5	4.4
231	678	799	BS IV	Gasoline	22.7	104.5	4.4
232	680	999	BS IV	Gasoline	23.0	103.1	4.3
233	850	998	BS IV	Gasoline	23.1	102.7	4.3
234	845	998	BS IV	Gasoline	23.1	102.7	4.3
235	840	998	BS IV	Gasoline	23.1	102.7	4.3
236	835	998	BS IV	Gasoline	23.1	102.7	4.3
237	815	998	BS IV	Gasoline	23.1	102.7	4.3
238	727	796	BS IV	Gasoline	24.0	99.0	4.2
239	722	796	BS IV	Gasoline	24.0	99.0	4.2
240	757	998	BS IV	Gasoline	24.1	98.5	4.1
241	755	998	BS IV	Gasoline	24.1	98.5	4.1
242	697	796	BS IV	Gasoline	24.7	96.0	4.0
243	2,840	4,461	BS IV	Diesel	8.6	275.0	11.6
244	2,740	4,461	BS IV	Diesel	8.9	267.0	11.2
245	2,030	2,149	BS IV	Diesel	12.5	250.0	8.0
246	2,011	2,596	BS IV	Diesel	12.4	250.0	8.1
247	2,394	3,198	BS IV	Diesel	10.6	249.0	9.4
248	2,375	2,982	BS IV	Diesel	10.2	233.0	9.8
249	2,495	2,987	BS IV	Diesel	11.5	230.0	8.7
250	2,150	2,157	BS IV	Diesel	11.5	230.0	8.7
251	2,175	2,179	BS IV	Diesel	11.8	225.0	8.5
252	1,920	2,179	BS IV	Diesel	12.2	216.8	8.2
253	2,280	2,179	BS IV	Diesel	12.3	216.0	8.1
254	2,105	2,999	BS IV	Diesel	12.3	215.5	8.1
255	2,010	2,999	BS IV	Diesel	12.3	215.5	8.1
256	1,964	2,596	BS IV	Diesel	12.4	211.6	8.1
257	2,110	2,179	BS IV	Diesel	12.6	211.0	7.9
258	1,954	2,143	BS IV	Diesel	12.6	210.0	7.9
259	2,238	2,198	BS IV	Diesel	12.6	209.5	7.9
260	1,945	2,499	BS IV	Diesel	12.7	209.0	7.9
261	1,935	2,499	BS IV	Diesel	12.7	209.0	7.9
262	1,685	2,489	BS IV	Diesel	13.0	204.3	7.7
263	1,845	2,179	BS IV	Diesel	13.1	202.3	7.6
264	2,445	2,993	BS IV	Diesel	13.2	200.8	7.6
265	2,396	2,993	BS IV	Diesel	13.2	200.8	7.6

Saudi Aramco: Public

266	2,353	2,993	BS IV	Diesel	13.2	200.8	7.6
267	2,343	2,993	BS IV	Diesel	13.2	200.8	7.6
268	2,010	2,179	BS IV	Diesel	13.2	200.8	7.6
269	2,234	2,993	BS IV	Diesel	13.4	198.0	7.5
270	2,292	2,198	BS IV	Diesel	13.6	194.9	7.4
271	2,295	2,987	BS IV	Diesel	13.7	193.0	7.3
272	1,920	2,179	BS IV	Diesel	13.8	192.6	7.2
273	1,989	2,993	BS IV	Diesel	13.4	191.3	7.5
274	2,015	2,179	BS IV	Diesel	13.9	191.0	7.2
275	2,000	2,179	BS IV	Diesel	13.9	191.0	7.2
276	2,029	1,999	BS IV	Diesel	14.1	188.7	7.1
277	2,024	1,999	BS IV	Diesel	14.1	188.7	7.1
278	2,055	1,999	BS IV	Diesel	14.3	188.3	7.0
279	2,036	1,999	BS IV	Diesel	14.3	188.3	7.0
280	2,032	1,999	BS IV	Diesel	14.3	188.3	7.0
281	1,790	2,489	BS IV	Diesel	14.1	188.0	7.1
282	1,800	2,179	BS IV	Diesel	14.1	187.7	7.1
283	2,240	2,143	BS IV	Diesel	14.2	187.0	7.0
284	2,204	2,198	BS IV	Diesel	14.2	187.0	7.0
285	2,155	2,755	BS IV	Diesel	12.7	187.0	7.9
286	2,155	2,755	BS IV	Diesel	12.7	187.0	7.9
287	2,155	2,755	BS IV	Diesel	12.7	187.0	7.9
288	1,940	2,179	BS IV	Diesel	14.2	187.0	7.0
289	2,322	2,993	BS IV	Diesel	14.1	186.7	7.1
290	2,302	2,993	BS IV	Diesel	14.1	186.7	7.1
291	2,279	2,993	BS IV	Diesel	14.1	186.7	7.1
292	2,202	2,993	BS IV	Diesel	14.1	186.7	7.1
293	2,155	2,755	BS IV	Diesel	12.7	186.0	7.9
294	2,155	2,755	BS IV	Diesel	12.7	186.0	7.9
295	1,890	2,755	BS IV	Diesel	12.8	185.0	7.8
296	1,890	2,755	BS IV	Diesel	12.8	185.0	7.8
297	1,890	2,755	BS IV	Diesel	12.8	185.0	7.8
298	2,181	2,179	BS IV	Diesel	14.5	183.0	6.9
299	1,925	2,179	BS IV	Diesel	14.5	182.6	6.9
300	2,083	2,179	BS IV	Diesel	14.6	181.0	6.8
301	1,905	2,993	BS IV	Diesel	14.7	180.0	6.8
302	1,830	2,523	BS IV	Diesel	14.8	178.4	6.8
303	1,913	1,999	BS IV	Diesel	15.2	177.5	6.6
304	1,913	1,999	BS IV	Diesel	15.2	177.5	6.6
305	1,901	1,999	BS IV	Diesel	15.2	177.5	6.6
306	1,901	1,999	BS IV	Diesel	15.2	177.5	6.6
307	1,900	1,999	BS IV	Diesel	15.2	177.5	6.6
308	1,900	1,999	BS IV	Diesel	15.2	177.5	6.6

309	1,857	1,999	BS IV	Diesel	15.2	177.5	6.6
310	1,865	1,999	BS IV	Diesel	15.7	176.9	6.4
311	1,857	1,999	BS IV	Diesel	15.7	176.9	6.4
312	1,832	1,999	BS IV	Diesel	15.7	176.9	6.4
313	1,803	1,999	BS IV	Diesel	15.7	176.9	6.4
314	1,825	2,179	BS IV	Diesel	15.0	176.6	6.7
315	1,825	2,179	BS IV	Diesel	15.0	176.5	6.7
316	1,735	2,197	BS IV	Diesel	15.0	176.2	6.7
317	1,913	1,999	BS IV	Diesel	16.4	175.5	6.1
318	1,875	2,393	BS IV	Diesel	13.6	174.0	7.4
319	1,875	2,393	BS IV	Diesel	13.6	174.0	7.4
320	1,710	2,523	BS IV	Diesel	15.2	173.7	6.6
321	1,710	2,197	BS IV	Diesel	15.3	173.3	6.5
322	1,855	2,393	BS IV	Diesel	13.7	173.0	7.3
323	1,855	2,393	BS IV	Diesel	13.7	173.0	7.3
324	1,855	2,393	BS IV	Diesel	13.7	173.0	7.3
325	1,855	2,393	BS IV	Diesel	13.7	173.0	7.3
326	1,855	2,393	BS IV	Diesel	13.7	173.0	7.3
327	1,855	2,393	BS IV	Diesel	13.7	173.0	7.3
328	1,855	2,393	BS IV	Diesel	13.7	173.0	7.3
329	1,855	2,393	BS IV	Diesel	13.7	173.0	7.3
330	1,855	2,393	BS IV	Diesel	13.7	173.0	7.3
331	1,855	2,393	BS IV	Diesel	13.7	173.0	7.3
332	1,490	2,523	BS IV	Diesel	15.4	172.4	6.5
333	1,845	2,179	BS IV	Diesel	15.4	172.0	6.5
334	1,940	2,987	BS IV	Diesel	15.6	170.0	6.4
335	1,640	1,493	BS IV	Diesel	15.6	170.0	6.4
336	1,723	1,995	BS IV	Diesel	15.8	168.6	6.3
337	2,150	2,925	BS VI	Diesel	16.1	165.0	6.2
338	1,863	1,995	BS IV	Diesel	16.1	165.0	6.2
339	1,510	1,493	BS IV	Diesel	16.2	164.0	6.2
340	1,641	1,956	BS IV	Diesel	16.3	162.5	6.1
341	1,880	1,950	BS VI	Diesel	16.5	161.0	6.1
342	1,656	1,995	BS IV	Diesel	16.4	160.5	6.1
343	1,826	1,968	BS IV	Diesel	16.6	160.0	6.0
344	1,675	1,956	BS IV	Diesel	16.6	160.0	6.0
345	1,720	1,968	BS IV	Diesel	14.6	159.0	6.8
346	1,946	2,993	BS IV	Diesel	16.8	158.0	6.0
347	1,860	1,995	BS IV	Diesel	16.8	158.0	6.0
348	1,620	1,493	BS IV	Diesel	16.8	157.3	6.0
349	1,410	2,523	BS IV	Diesel	17.0	155.6	5.9
350	1,990	2,993	BS IV	Diesel	17.1	155.0	5.8
351	1,880	1,950	BS VI	Diesel	17.1	155.0	5.8

352	1,537	1,956	BS IV	Diesel	17.1	154.7	5.8
353	1,650	1,497	BS IV	Diesel	17.3	154.2	5.8
354	1,610	1,493	BS IV	Diesel	17.3	153.2	5.8
355	1,776	1,999	BS IV	Diesel	19.3	152.1	5.2
356	1,747	1,999	BS IV	Diesel	19.3	152.1	5.2
357	1,704	1,999	BS IV	Diesel	19.3	152.1	5.2
358	1,848	2,993	BS IV	Diesel	17.4	152.0	5.7
359	1,550	1,968	BS IV	Diesel	17.4	152.0	5.7
360	1,423	1,582	BS IV	Diesel	17.6	150.5	5.7
361	1,170	909	BS IV	Diesel	17.7	149.4	5.6
362	1,715	1,950	BS VI	Diesel	17.8	149.0	5.6
363	1,408	1,582	BS IV	Diesel	18.2	145.0	5.5
364	1,170	798	BS IV	Diesel	18.3	145.0	5.5
365	1,151	909	BS IV	Diesel	18.3	144.8	5.5
366	1,725	1,597	BS IV	Diesel	18.3	144.7	5.5
367	1,614	1,995	BS IV	Diesel	18.4	142.6	5.4
368	1,910	1,995	BS IV	Diesel	18.6	142.0	5.4
369	1,656	2,143	BS IV	Diesel	18.7	142.0	5.3
370	1,565	1,968	BS IV	Diesel	18.7	142.0	5.3
371	1,075	798	BS IV	Diesel	18.7	142.0	5.3
372	1,677	1,950	BS VI	Diesel	18.8	141.0	5.3
373	1,225	909	BS IV	Diesel	19.0	139.4	5.3
374	1,690	1,995	BS IV	Diesel	19.2	138.0	5.2
375	1,625	1,995	BS IV	Diesel	19.2	138.0	5.2
376	1,572	1,995	BS IV	Diesel	19.2	138.0	5.2
377	1,585	2,143	BS IV	Diesel	19.3	137.0	5.2
378	1,000	702	BS IV	Diesel	19.3	137.0	5.2
379	1,387	1,461	BS IV	Diesel	19.4	136.6	5.2
380	1,713	1,995	BS IV	Diesel	19.5	136.0	5.1
381	1,395	1,968	BS IV	Diesel	19.5	136.0	5.1
382	1,666	1,597	BS IV	Diesel	19.5	135.8	5.1
383	1,643	1,999	BS IV	Diesel	20.5	135.5	4.9
384	1,638	1,999	BS IV	Diesel	20.5	135.5	4.9
385	1,609	1,999	BS IV	Diesel	20.5	135.5	4.9
386	1,368	1,497	BS IV	Diesel	19.6	135.1	5.1
387	1,580	1,995	BS IV	Diesel	19.6	135.0	5.1
388	1,570	2,143	BS IV	Diesel	19.6	135.0	5.1
389	1,305	1,497	BS IV	Diesel	19.6	135.0	5.1
390	1,305	1,497	BS IV	Diesel	19.6	135.0	5.1
391	1,153	1,498	BS IV	Diesel	19.6	135.0	5.1
392	1,421	1,461	BS IV	Diesel	19.7	134.3	5.1
393	1,326	1,461	BS IV	Diesel	19.8	134.0	5.1
394	1,210	909	BS IV	Diesel	19.9	133.2	5.0

Saudi Aramco: Public

395	1,375	1,461	BS IV	Diesel	19.9	133.0	5.0
396	1,216	1,598	BS IV	Diesel	19.9	133.0	5.0
397	1,404	1,497	BS IV	Diesel	20.0	132.4	5.0
398	1,321	1,461	BS IV	Diesel	20.0	132.4	5.0
399	1,287	1,461	BS IV	Diesel	20.0	132.4	5.0
400	1,185	1,248	BS IV	Diesel	20.1	132.0	5.0
401	1,170	1,248	BS IV	Diesel	20.1	132.0	5.0
402	1,368	1,461	BS IV	Diesel	20.1	131.5	5.0
403	1,238	1,598	BS IV	Diesel	20.2	131.0	5.0
404	1,679	1,995	BS IV	Diesel	20.4	130.0	4.9
405	1,349	1,461	BS IV	Diesel	20.4	130.0	4.9
406	1,158	1,498	BS IV	Diesel	20.4	130.0	4.9
407	1,358	1,461	BS IV	Diesel	20.5	129.5	4.9
408	1,398	1,582	BS IV	Diesel	20.5	129.2	4.9
409	1,163	1,498	BS IV	Diesel	20.5	129.0	4.9
410	1,337	1,496	BS IV	Diesel	20.7	128.0	4.8
411	1,232	1,496	BS IV	Diesel	20.7	128.0	4.8
412	1,130	1,047	BS IV	Diesel	20.9	127.0	4.8
413	1,340	1,968	BS IV	Diesel	21.0	126.0	4.8
414	1,255	1,582	BS IV	Diesel	21.0	126.0	4.8
415	1,354	1,461	BS IV	Diesel	21.2	125.1	4.7
416	1,270	1,364	BS IV	Diesel	19.1	124.0	5.2
417	1,270	1,364	BS IV	Diesel	19.1	124.0	5.2
418	1,270	1,364	BS IV	Diesel	19.1	124.0	5.2
419	1,210	1,498	BS IV	Diesel	21.4	124.0	4.7
420	1,150	1,461	BS IV	Diesel	21.5	123.4	4.7
421	1,085	1,047	BS IV	Diesel	21.5	123.0	4.7
422	1,228	1,498	BS IV	Diesel	21.7	122.0	4.6
423	1,170	1,198	BS IV	Diesel	21.7	122.0	4.6
424	1,155	1,248	BS IV	Diesel	21.7	122.0	4.6
425	1,154	1,248	BS IV	Diesel	21.7	122.0	4.6
426	1,184	1,498	BS IV	Diesel	21.8	121.3	4.6
427	1,203	1,396	BS IV	Diesel	21.7	121.0	4.6
428	1,306	1,498	BS IV	Diesel	21.9	120.9	4.6
429	1,346	1,396	BS IV	Diesel	22.1	119.8	4.5
430	1,213	1,396	BS IV	Diesel	22.2	119.4	4.5
431	1,369	1,582	BS IV	Diesel	22.5	116.9	4.4
432	1,151	1,396	BS IV	Diesel	22.5	116.7	4.4
433	1,116	1,461	BS IV	Diesel	22.8	116.1	4.4
434	1,302	1,498	BS IV	Diesel	23.0	115.2	4.3
435	1,000	1,461	BS IV	Diesel	23.2	114.2	4.3
436	1,040	1,364	BS IV	Diesel	21.2	112.0	4.7
437	1,040	1,364	BS IV	Diesel	21.2	112.0	4.7

438	1,040	1,364	BS IV	Diesel	21.2	112.0	4.7
439	1,040	1,364	BS IV	Diesel	21.2	112.0	4.7
440	1,040	1,364	BS IV	Diesel	21.2	112.0	4.7
441	1,040	1,364	BS IV	Diesel	21.2	112.0	4.7
442	1,040	1,364	BS IV	Diesel	21.2	112.0	4.7
443	1,040	1,364	BS IV	Diesel	21.2	112.0	4.7
444	1,040	1,364	BS IV	Diesel	21.2	112.0	4.7
445	1,040	1,364	BS IV	Diesel	21.2	112.0	4.7
446	1,040	1,364	BS IV	Diesel	21.2	112.0	4.7
447	1,040	1,364	BS IV	Diesel	21.2	112.0	4.7
448	1,040	1,364	BS IV	Diesel	21.2	112.0	4.7
449	1,040	1,364	BS IV	Diesel	21.2	112.0	4.7
450	1,040	1,498	BS IV	Diesel	23.8	111.3	4.2
451	1,025	1,120	BS IV	Diesel	24.0	110.0	4.2
452	1,046	1,120	BS IV	Diesel	24.1	109.7	4.1
453	1,210	1,248	BS IV	Diesel	24.3	109.0	4.1
454	1,200	1,248	BS IV	Diesel	24.3	109.0	4.1
455	1,089	1,498	BS IV	Diesel	24.4	108.5	4.1
456	1,228	1,582	BS IV	Diesel	24.8	107.0	4.0
457	1,093	1,186	BS IV	Diesel	24.8	106.0	4.0
458	1,240	1,248	BS IV	Diesel	25.1	105.5	4.0
459	1,175	1,498	BS IV	Diesel	25.1	105.5	4.0
460	1,205	1,396	BS IV	Diesel	25.2	105.0	4.0
461	1,108	1,186	BS IV	Diesel	25.1	105.0	4.0
462	1,056	1,498	BS IV	Diesel	25.5	104.0	3.9
463	1,204	1,498	BS IV	Diesel	25.5	103.9	3.9
464	1,148	1,498	BS IV	Diesel	25.6	103.4	3.9
465	1,081	1,498	BS IV	Diesel	26.1	101.5	3.8
466	1,045	1,248	BS IV	Diesel	26.6	99.6	3.8
467	1,353	1,597	BS IV	Diesel	26.8	98.8	3.7
468	970	1,248	BS IV	Diesel	26.8	98.8	3.7
469	960	1,248	BS IV	Diesel	26.8	98.8	3.7
470	1,125	1,498	BS IV	Diesel	26.8	98.7	3.7
471	1,155	1,498	BS IV	Diesel	27.3	97.0	3.7
472	1,023	1,498	BS IV	Diesel	27.4	96.7	3.6
473	985	1,248	BS IV	Diesel	27.4	96.7	3.6
474	985	1,248	BS IV	Diesel	27.4	96.7	3.6
475	990	1,248	BS IV	Diesel	28.4	93.3	3.5
476	990	1,248	BS IV	Diesel	28.4	93.3	3.5
477	985	1,248	BS IV	Diesel	28.4	93.3	3.5
478	980	1,248	BS IV	Diesel	28.4	93.3	3.5

Supplementary Table 2 Raw data on tailpipe emissions and fuel economy of 131 passenger road 2W vehicles in India in 2018/19 [1].

Count	Class	Engine size (cm^3)	Fuel	Fuel Economy (km/L)	Tailpipe CO_2 (g/km)	Fuel Consumption (L/100km)
1	Class 1	88	Gasoline	59.6	38.9	1.7
2	Class 1	97	Gasoline	67.2	35.3	1.5
3	Class 1	97	Gasoline	67.2	35.3	1.5
4	Class 1	97	Gasoline	67.2	35.3	1.5
5	Class 1	97	Gasoline	67.2	35.3	1.5
6	Class 1	97	Gasoline	67.2	35.3	1.5
7	Class 1	97	Gasoline	67.2	35.3	1.5
8	Class 1	100	Gasoline	60.2	38.5	1.7
9	Class 1	100	Gasoline	60.2	38.5	1.7
10	Class 1	100	Gasoline	60.2	38.5	1.7
11	Class 1	100	Gasoline	60.2	38.5	1.7
12	Class 1	100	Gasoline	70.2	33.0	1.4
13	Class 1	102	Gasoline	74.1	30.1	1.3
14	Class 1	102	Gasoline	74.1	30.1	1.3
15	Class 1	102	Gasoline	82.6	27.6	1.2
16	Class 1	102	Gasoline	49.4	48.0	2.0
17	Class 1	107	Gasoline	62.1	36.3	1.6
18	Class 1	109	Gasoline	68.3	34.2	1.5
19	Class 1	109	Gasoline	68.3	34.2	1.5
20	Class 1	109	Gasoline	68.3	34.2	1.5
21	Class 1	109	Gasoline	54.2	43.1	1.8
22	Class 1	109	Gasoline	53.4	43.9	1.9
23	Class 1	109	Gasoline	54.4	42.8	1.8
24	Class 1	109	Gasoline	55.5	42.2	1.8
25	Class 1	109	Gasoline	53.5	43.4	1.9
26	Class 1	109	Gasoline	51.8	44.9	1.9
27	Class 1	109	Gasoline	76.2	29.1	1.3
28	Class 1	109	Gasoline	65.5	33.8	1.5
29	Class 1	109	Gasoline	69.7	31.9	1.4
30	Class 1	110	Gasoline	48.5	48.1	2.1
31	Class 1	110	Gasoline	53.2	43.6	1.9
32	Class 1	110	Gasoline	54.5	42.5	1.8
33	Class 1	110	Gasoline	54.5	42.5	1.8
34	Class 1	110	Gasoline	54.5	42.5	1.8
35	Class 1	110	Gasoline	54.5	42.5	1.8
36	Class 1	110	Gasoline	54.5	42.5	1.8
37	Class 1	110	Gasoline	54.5	42.5	1.8
38	Class 1	110	Gasoline	65.4	35.4	1.5

Saudi Aramco: Public

39	Class 1	110	Gasoline	65.8	35.2	1.5
40	Class 1	110	Gasoline	69.3	33.4	1.4
41	Class 1	110	Gasoline	77.4	30.3	1.3
42	Class 1	111	Gasoline	51.5	45.8	1.9
43	Class 1	111	Gasoline	49.5	47.8	2.0
44	Class 1	113	Gasoline	50.0	47.0	2.0
45	Class 1	113	Gasoline	50.0	47.0	2.0
46	Class 1	113	Gasoline	50.0	47.0	2.0
47	Class 1	113	Gasoline	50.0	47.0	2.0
48	Class 1	113	Gasoline	50.0	47.0	2.0
49	Class 1	116	Gasoline	80.6	28.5	1.2
50	Class 1	116	Gasoline	84.0	27.1	1.2
51	Class 1	116	Gasoline	76.3	29.5	1.3
52	Class 1	124	Gasoline	57.1	40.4	1.8
53	Class 1	124	Gasoline	56.9	40.3	1.8
54	Class 1	125	Gasoline	66.7	33.9	1.5
55	Class 1	125	Gasoline	51.5	45.2	1.9
56	Class 1	125	Gasoline	47.8	48.3	2.1
57	Class 1	125	Gasoline	41.0	54.4	2.4
58	Class 1	125	Gasoline	58.9	39.7	1.7
59	Class 1	125	Gasoline	58.9	39.7	1.7
60	Class 1	125	Gasoline	62.2	37.5	1.6
61	Class 1	125	Gasoline	64.3	36.7	1.6
62	Class 1	125	Gasoline	64.3	36.7	1.6
63	Class 1	125	Gasoline	64.2	36.1	1.6
64	Class 1	125	Gasoline	62.6	36.6	1.6
65	Class 1	125	Gasoline	49.2	47.1	2.0
66	Class 1	125	Gasoline	49.0	47.8	2.0
67	Class 1	125	Gasoline	51.0	44.7	2.0
68	Class 1	125	Gasoline	60.8	38.6	1.6
69	Class 2-1	149	Gasoline	51.9	43.8	1.9
70	Class 2-1	149	Gasoline	51.9	43.8	1.9
71	Class 2-1	149	Gasoline	50.9	45.7	2.0
72	Class 2-1	149	Gasoline	50.4	47.2	2.0
73	Class 2-1	149	Gasoline	55.5	42.5	1.8
74	Class 2-1	149	Gasoline	54.6	42.4	1.8
75	Class 2-1	150	Gasoline	50.3	45.4	2.0
76	Class 2-1	155	Gasoline	53.6	43.4	1.9
77	Class 2-1	155	Gasoline	49.0	47.4	2.0
78	Class 2-1	155	Gasoline	49.5	46.5	2.0
79	Class 2-2	155	Gasoline	50.1	45.9	2.0
80	Class 2-2	155	Gasoline	49.6	45.6	2.0
81	Class 2-1	160	Gasoline	46.9	49.4	2.1

82	Class 2-1	160	Gasoline	45.2	51.3	2.2
83	Class 2-1	160	Gasoline	46.3	50.1	2.2
84	Class 2-1	160	Gasoline	46.5	49.2	2.2
85	Class 2-1	160	Gasoline	43.5	53.2	2.3
86	Class 2-1	163	Gasoline	51.7	44.1	1.9
87	Class 2-1	163	Gasoline	57.3	40.3	1.7
88	Class 2-1	177	Gasoline	43.3	53.5	2.3
89	Class 2-1	179	Gasoline	47.4	49.1	2.1
90	Class 2-2	198	Gasoline	40.2	57.7	2.5
91	Class 2-2	198	Gasoline	44.8	51.7	2.2
92	Class 2-2	200	Gasoline	37.9	60.7	2.6
93	Class 2-2	200	Gasoline	40.3	57.0	2.5
94	Class 2-2	200	Gasoline	37.9	60.8	2.6
95	Class 2-2	200	Gasoline	39.4	57.7	2.5
96	Class 2-1	200	Gasoline	44.4	52.5	2.3
97	Class 2-2	220	Gasoline	41.8	54.7	2.4
98	Class 2-1	220	Gasoline	43.5	53.4	2.3
99	Class 2-1	220	Gasoline	43.5	53.4	2.3
100	Class 3-2	249	Gasoline	33.2	69.5	3.0
101	Class 2-2	249	Gasoline	42.1	54.6	2.4
102	Class 2-2	249	Gasoline	42.1	54.6	2.4
103	Class 3-1	249	Gasoline	38.6	60.8	2.6
104	Class 2-2	250	Gasoline	38.1	60.0	2.6
105	Class 3-1	286	Gasoline	37.2	63.4	2.7
106	Class 3-1	295	Gasoline	28.9	79.7	3.5
107	Class 2-2	295	Gasoline	33.7	68.2	3.0
108	Class 3-2	312	Gasoline	30.6	75.8	3.3
109	Class 3-2	321	Gasoline	24.9	97.9	4.0
110	Class 2-1	346	Gasoline	40.8	56.9	2.5
111	Class 2-1	346	Gasoline	40.8	56.9	2.5
112	Class 2-1	346	Gasoline	40.8	56.9	2.5
113	Class 2-1	346	Gasoline	40.8	56.9	2.5
114	Class 2-1	346	Gasoline	40.8	56.9	2.5
115	Class 2-1	346	Gasoline	40.8	56.9	2.5
116	Class 3-2	373	Gasoline	28.6	81.3	3.5
117	Class 3-2	373	Gasoline	30.0	77.6	3.3
118	Class 3-2	373	Gasoline	29.2	79.1	3.4
119	Class 2-2	411	Gasoline	37.3	62.5	2.7
120	Class 2-2	499	Gasoline	35.8	64.6	2.8
121	Class 2-2	499	Gasoline	35.8	64.6	2.8
122	Class 2-2	499	Gasoline	35.8	64.6	2.8
123	Class 2-2	499	Gasoline	35.8	64.6	2.8
124	Class 2-2	499	Gasoline	35.8	64.6	2.8

125	Class 3-2	645	Gasoline	25.2	92.8	4.0
126	Class 3-2	648	Gasoline	25.7	92.3	3.9
127	Class 3-2	648	Gasoline	25.7	92.3	3.9
128	Class 3-2	649	Gasoline	23.3	101.3	4.3
129	Class 3-2	749	Gasoline	22.0	112.4	4.5
130	Class 3-2	999	Gasoline	23.5	103.7	4.3
131	Class 3-2	1340	Gasoline	18.0	137.3	5.6

Supplementary References

[1] SIAM, "SIAM FUEL EFFICIENCY DATA," Society of Indian Automobile Manufacturers , https://www.siam.in/cpage.aspx?mpgid=31&pgidtrail=82.

[2] CEA, "Data from the Central Electricity Authority, Ministry of Power, Government of India," [Online]. Available: https://cea.nic.in/?lang=en.

[3] CEA, "REPORT ON OPTIMAL GENERATION CAPACITY MIX FOR 2029-30," Central Electricity Authority (CEA), Ministry of Power, Government of India, 2020.

[4] K. Surana and S. M. Jordaan, "The climate mitigation opportunity behind global power transmission and distribution," *Nature Climate Change,* vol. 9, no. https://doi.org/10.1038/s41558-019-0544-3, pp. 660-665, 2019.

[5] IEA, "Electricity Market Report - July 2021," International Energy Agency, 2021.

[6] Sphera, "GaBi version 9.2 - LCA software and 2020 Life Cycle Inventory databases," Sphera (formerly thinkstep), 2020.

[7] SIAM, "Society of Indian Automobile Manufacturers. Annual Report 2018-19. Building the nation responsibly.," Society of Indian Automobile Manufacturers, 2019.

[8] A. Soman, H. Kaur, H. Jain and K. Ganesan, "India's Electric Vehicle Transition. Can Electric Mobility Support India's Sustainable Economic Recovery Post COVID-19?," Council on Energy, Environment and Water (CEEW), New Delhi, 2020.

[9] D. Wu, F. Guo, F. R. Field III, R. D. De Kleine, H. C. Kim, T. J. Wallington and R. E. Kirchain, "Regional Heterogeneity in the Emissions Benefits of Electrified and Lightweighted Light-Duty Vehicles," *Environmental Science & Technology,* vol. 53, no. 18, p. 10560, 2019.

[10] GOI, "Open Government Data (OGD) Platform India," National Informatics Centre (NIC), Ministry of Electronics & Information Technology, Government of India, [Online]. Available: https://data.gov.in/resources/monthly-seasonal-and-annual-mean-temp-series-1901-2017. [Accessed 10 September 2020].

[11] B. L. Cox and C. L. Mutel, "The environmental and cost performance of current and future motorcycles," *Applied Energy,* vol. 212, no. https://doi.org/10.1016/j.apenergy.2017.12.100, pp. 1013-1024, 2018.

[12] ICCT, "Fuel consumption of new passenger cars in India: Manufacturers' performance in fiscal year 2018–19," INTERNATIONAL COUNCIL ON CLEAN TRANSPORTATION, 2020.

Saudi Aramco: Public
